# fMRI biomarkers of social cognitive skills training in psychosis: Extrinsic and intrinsic functional connectivity

**DOI:** 10.1371/journal.pone.0214303

**Published:** 2019-05-17

**Authors:** Junghee Lee, Amy M. Jimenez, William P. Horan, Michael F. Green

**Affiliations:** 1 Department of Psychiatry and Biobehavioral Sciences, David Geffen School of Medicine at University of California Los Angeles, Los Angeles, CA, United States of America; 2 Desert Pacific Mental Illness Research, Education and Clinical Center, Veterans Affairs Greater Los Angeles Healthcare System, Los Angeles, CA, United States of America; University of Lausanne, SWITZERLAND

## Abstract

Social cognitive skills training interventions for psychotic disorders have shown improvement in social cognitive performance tasks, but little was known about brain-based biomarkers linked to treatment effects. In this pilot study, we examined whether social cognitive skills training could modulate extrinsic and intrinsic functional connectivity in psychosis using functional magnetic resonance imaging (fMRI). Twenty-six chronic outpatients with psychotic disorders were recruited from either a Social Cognitive Skills Training (SCST) or an activity- and time-matched control intervention. At baseline and the end of intervention (12 weeks), participants completed two social cognitive tasks: a Facial Affect Matching task and a Mental State Attribution Task, as well as resting-state fMRI (rs-fMRI). Extrinsic functional connectivity was assessed using psychophysiological interaction (PPI) with amygdala and temporo-parietal junction as a seed region for the Facial Affect Matching Task and the Mental State Attribution task, respectively. Intrinsic functional connectivity was assessed with independent component analysis on rs-fMRI, with a focus on the default mode network (DMN). During the Facial Affect Matching task, we observed stronger PPI connectivity in the SCST group after intervention (compared to baseline), but no treatment-related change in the Control group. Neither group showed treatment-related changes in PPI connectivity during the Mental State Attribution task. During rs-fMRI, we found treatment-related changes in the DMN in the SCST group, but not in Control group. This study found that social cognitive skills training modulated both extrinsic and intrinsic functional connectivity in individuals with psychotic disorders after a 12-week intervention. These findings suggest treatment-related changes in functional connectivity as a potential brain-based biomarker of social cognitive skills training.

## Introduction

Social cognitive impairment is a core feature of psychosis and closely related to poor social functioning. Pharmacological intervention has shown limited efficacy, but findings from skills training interventions have been encouraging in that they show improvement on some social cognitive domains in psychosis, including facial affect perception and mental state attribution [1, 2]. However, most studies on training intervention have employed performance-based assessments [1, 2]; few studies have examined brain-based biomarkers of social cognitive interventions in psychosis. The lack of a validated biomarker clearly limits a mechanistic understanding of the efficacy of training interventions on social cognitive impairment.

Considerable work has been done to understand the neural underpinnings of social processing in healthy individuals [3, 4]. In addition to identifying key brain regions, emerging evidence highlights the importance of the functional connectivity among brain regions for social processes [5, 6]. Thus, it is not surprising that there is now growing interest in the connectivity among brain regions in research in psychosis [7].

One way to examine connections among nodes is with extrinsic functional connectivity (i.e., task-based functional connectivity) during social cognitive tasks. For example, during facial affect recognition tasks, schizophrenia patients have shown aberrant functional connectivity of amygdala with medial prefrontal cortex (mPFC) [8, 9]. During mental state attribution tasks, patients have shown reduced connectivity involving the temporo-parietal junction [10]. Another way to examine connections is with intrinsic functional connectivity (i.e., resting-state connectivity such as the default mode network [DMN]) [11]. The DMN is of particular interest in this regard because it is heavily overlapping with the network involved with mental state attribution [12]. The DMN generally includes midline regions within posterior cingulate cortex, precuneus, mPFC, and bilateral temporo-parietal junction [11]. Thus, it has been suggested that schizophrenia patients may have aberrant connectivity related to DMN and this abnormal pattern could be associated with their impaired social functioning. While only a few studies have directly examined this possibility, existing evidence largely supports the potential importance of DMN in social functioning in schizophrenia. For example, schizophrenia patients have shown altered patterns of intrinsic connectivity within DMN compared to controls. The altered pattern within DMN was associated with impaired social functioning among schizophrenia patients [13], as well as among individuals at familial high-risk for schizophrenia [14].

It remains unknown as to whether extrinsic or intrinsic functional connectivity could be modulated as a function of training on social cognitive processes in psychosis. Hence, this pilot study examined the feasibility of using functional connectivity-based biomarkers of a social cognitive intervention in people with psychotic disorders. Extrinsic functional connectivity was examined using a psychophysiological interaction approach (PPI) [15] during two social cognitive tasks, a Facial Affect Matching task and a Mental State Attribution task. PPI is a powerful tool to examine the pattern of connectivity between a seed region of interests and other brain regions during a particular task in the scanner. Intrinsic functional connectivity focused on the treatment-related changes in DMN. Subjects were assessed at baseline and after 12 weeks of a social cognitive intervention program or a time-matched control.

## Materials and methods

### Study design and participants

Twenty-six chronic outpatients were recruited from a larger randomized controlled trial examining the efficacy of the Social Cognitive Skills Training (SCST) program that our research group previously developed (Clinical Trials #NCT01267019). The data collection for the current paper was supported by NARSAD Young Investigator Grant (to JL) that was aimed to collect fMRI data on a subset of the training cohorts to assess the treatment-related effects of the SCST on extrinsic and intrinsic functional connectivity in individuals with psychotic disorders. The parent study had three arms (“SCST-in vivo”, “SCST-Clinic”, and “Control”), but given the limited budget of the NARSAD grant, this fMRI sub-study recruited participants from only two of those arms: SCST-Clinic and Control (herein referred to as SCST and Control). Details of the parent study are provided elsewhere [16]. Both the SCST and the control condition consisted for 30 sessions over a 12 week period. Participants underwent fMRI procedures at baseline (0 wk) and end-point (12 wk).

All participants met DSM-IV criteria for schizophrenia, schizoaffective disorder, or psychosis NOS. The diagnostic eligibility was determined by medical records and consultation with treating psychiatrists. All patients were clinically stable (i.e., no psychiatric hospitalization in the past 2 months, no change of antipsychotic medication for the past 6 weeks). Selection criteria for participants were: age 18–60; no current or past neurological disorder (e.g., epilepsy); no history of serious head injury (i.e., loss of consciousness > 60 mins); IQ<70; no alcohol or substance dependence in the last 6 months; and no women who are pregnant or think they might be pregnant based on self-report; and no contraindications for fMRI scan (e.g., metal in body).

All participants were evaluated for the capacity to give informed consent and provided written informed consents after procedures were fully explained, as approved by the Institutional Review Boards at University of California Los Angeles and the Veterans Affairs Greater Los Angeles Healthcare System.

### Interventions

Details of the interventions are provided elsewhere [16]. Briefly, the SCST intervention included five modules: emotion processing, social perception, how emotions color our social interpretations, understanding others’ emotions, and understanding others’ intentions. The training approach incorporated several skills-building strategies with a variety of training stimuli (e.g., photos, audio clips, written vignettes, and video clips). Control intervention included 30 sessions of Illness management training. The Control intervention included materials on nutrition and relaxation and selections from the UCLA Social and Independent Living Skills Program [17].

### fMRI procedures

In the scanner, participants completed two tasks, a Facial Affect Matching task and a Mental State Attribution task, as well as a 5-minute resting fMRI (rsfMRI) scan. Extrinsic functional connectivity was examined using task-based fMRI data and intrinsic functional connectivity was examined using rsfMRI.

The Facial Affect Matching task was modified from a paradigm used in our previous study [18]. With a block design, this task consisted of two conditions that utilized the identical set of emotional face stimuli: emotion matching and gender matching. For each trial, a cue appeared for 2 seconds (emotion or gender); then, three stimuli appeared in a triangular arrangement for 3 seconds, one stimulus on the screen’s top (i.e., target), and two stimuli side-by-side at the bottom of the screen (i.e., the response choices). Participants were asked to indicate which stimulus at the bottom matches the target at the top according to the cue. There were 3 runs, each with 3 blocks of emotion, 3 blocks of emotion and 3 blocks of fixation. The primary contrast of interest was [emotion matching > gender matching], which was designed to separate neural activity related to facial affect recognition while controlling for neural activity related to non-affective judgments of face stimuli.

The Mental State Attribution Task [19] used an event-related design and consisted of three conditions that utilized written vignettes: false belief, false photograph and simple reading. In the false belief condition, subjects were asked to indicate what the beliefs of a character in the vignette would be, even when the beliefs differ from the actual state of affairs. The false photograph vignettes had the same story structure and required the same level of reasoning as the false belief condition, but lacked mental state attribution. The simple reading condition consisted of stories about nonhuman objects. For each trial, a brief vignette was presented for 12 sec, followed by a single, two alternative, forced-choice “fill-in-the-blank” question for 10 sec while the vignette was still visible. After the vignette and question was disappeared, a probe was presented for 3 sec, prompting the response. There were six runs, with six trials per run (two trails of each condition). The primary contrast of interest was [false belief > false photograph], which allowed us to separate neural activity related to mental state attribution while controlling for other task demands (e.g., reasoning).

### fMRI data acquisition and pre-processing

All MRI data were collected at the UCLA Staglin Center for Cognitive Neuroscience on a 3T Siemens Tim Trio scanner with a 12-channel head coil (Siemens Medical Solutions, Erlangen, Germany) using MR-compatible LCD goggles (Resonance Technology, Northridge, CA). Both paradigms were presented using E-prime software and behavioral performance was recorded using an MR-compatible 4-button response box (Resonance Technology, Northridge, CA). For anatomical reference, a high-resolution echo planar axial T2-weighted image (TR = 6000ms, TE = 66ms, flip angel = 90 degrees, voxel = 1.5x1.5x3, field of view = 192x192, 38 slices) and T1-weighted image with a Magnetization-Prepared Rapid Gradient Echo (MPRAGE) sequence (TR = 1900ms, TE = 3.4ms, flip angle = 9 degrees, voxel = 1x1x1mm, field of view = 256x256, 160 slices) were collected. To detect blood-oxygen-level-dependent (BOLD) signal, a T2*-weighted gradient-echo sequence was used (TR = 2500ms, TE = 35ms, flip angle = 75 degrees, voxel = 3x3x3mm, field of view = 64x64, 38 slices).

fMRI data preprocessing was carried out using FSL (version 5.0.9), the FMRIB Software Library (http://fsl.fmrib.ox.ac.uk/fsl). Preprocessing steps included high-pass filtering using a 100 s cut-off, spatial smoothing using a 5 mm full-width at half-maximum Gaussian kernel, non-brain removal using BET[20], and registration using FSL’s FLIRT (FMRIB’s Linear Image Registration Tool v6.0) [21]. fMRI data were registered first to the co-planar T2-weighted image (affine transformation; 6 degrees of freedom), then to the T1-weighted MPRAGE (Boundary-Based Registration, BBR) [22] and finally, to Montreal Neurological Institute (MNI) standard space (affine transformation, 12 degrees of freedom). To address potential motion artifacts, all images were realigned to the middle volume using MCFLIRT and movement parameters calculated by MCFLIRT were modeled as nuisance covariates [21, 23].

### fMRI data analyses

#### Extrinsic functional connectivity

Extrinsic functional connectivity was assessed using PPI [15] implemented in FSL for each paradigm. PPI allows us to identify extrinsic, task-dependent functional connectivity between a seed region and relevant brain regions as a function of task conditions. In other words, PPI enables us to examine which voxels of the brain changes fMRI activations along with a seed region of interest under a specific task context. Task-specific changes in the relationship between brain regions (i.e., a seed region of interest and other brain regions) indicates a task-specific changes in connectivity between regions. Motion parameters from MCFLIRT and the confounding matrix from the FSL Motion Outlier tool were included as nuisance variables to control for any potential effects of motion during the scan.

For the Facial Affect Matching task, we used amygdala as the seed region. The amygdala seed was created based on the HarvardOxford subcortical probability atlas. For each participant, the time series of the amygdala seed was entered into general linear model, and PPI connectivity between the seed region and the rest of the brain was examined in relation to the contrast of interest (i.e., [emotion matching > gender matching]). For the Mental State Attribution task, we focused on TPJ as the seed region. The TPJ seed was created using a 10-mm sphere around peak coordinates identified in previous studies (left TPJ, x = -52, y = -56, z = 24; right TPJ, x = 56, y = -54, z = 24) [24, 25]. The time series of the TPJ seed was entered into general linear model, and PPI connectivity between the seed region and the rest of brain was examined in relation to the contrast of interest (i.e., [false belief > false photograph]).

To examine within-group and between-group effects, a mixed-effect model (FLAME1) was employed for each paradigm. The resulting statistical images of PPI connectivity were thresholded using the cluster threshold of z>2.3 and p < .05, corrected for multiple comparison using Gaussian random field theory [26].

#### Intrinsic functional connectivity

For intrinsic functional connectivity, each participant’s smoothed, normalized rsfMRI images were subjected to group temporal concatenation and analyzed with FSL MELODIC Independent Component Analysis (ICA) software [27]. This method provides data-driven, unconstrained decomposition into statistically independent spatial components and their associated time series. From the group ICA results, we identified the component for the DMN using a published template of the DMN [28] to select the component with the best fit based on highest degree of spatial correlation. FSL’s “*fslcc*” tool was used to calculate Pearson’s *r* for each ICA component with the template image.

To investigate within and between group effects, the group ICA DMN component was used to estimate individual DMN maps and associated time-series using a dual regression approach implemented in FSL [29]. FSL’s “*randomise*,” nonparametric permutation tests (5000 permutations) were used to detect statistically significant effects using a family-wise error corrected significance threshold of p < .05 with threshold-free cluster enhancement [30, 31].

## Results

Table 1 showed the demographic and clinical characteristics, as well as attendance data of the participants. The two groups were comparable on age, gender, personal education, parental education, clinical symptoms, and session attendance. Performance data (see Table 2) indicates that all participants could perform the task above the chance level in the scanner. Relative motion parameters during each task and rsfMRI are presented in Table 2. For both tasks and rsfMRI, the two groups showed comparable motion during the scan at baseline and at 12 wks. For both groups, we also did not observe any difference on motion parameters between scans at baseline and 12 wks. Task-related activation at baseline across both groups are presented in the Supplement.

**Table 1:** Demographics, clinical characteristics and Attendance data of participants.

	SCST (n = 13)	Control (n = 13)	statistics
Age (yrs)	49.8 (7.7)	42.2 (12.2)	F_(1,24)_ = 3.60, p = .07
Gender (% female)	26.9	19.2	χ^2^ = .61, p = .42
Personal Edu. (yrs)	13.0 (2.8)	14.8 (1.9)	F_(1,24)_ = 2.06, p = .16
Parental Edu. (yrs)	15.0 (3.0)	14.0 (4.2)	F_(1,24)_ = .08, p = .54
Sessions attended	25.0 (5.6)	22.1 (2.9)	F_(1,24)_ = 2.47, p = .12
BPRS total			
Baseline	43.0 (10.6)	44.5 (16.1)	F_(1,24)_ = 0.7, p = .78
12 wk	38.3 (8.9)	46.3 (9.5)	F_(1,18)_ = 3.72, p = .07

Note: Values are presented as mean (SD).

**Table 2:** Performance data and relative motion in the scanner.

	SCST		Control	
	Baseline (n = 13)	12 wk (n = 11)	Baseline (n = 13)	12 wk (n = 11)
**Performance data**				
Facial Affect Matching task				
Emotion	.67 (.07)	.72 (.10)	.68 (.11)	.72 (.12)
Gender	.89 (.08)	.91 (.08)	.81 (.16)	.84 (.13)
Mental State Attribution task				
False belief	9.3 (1.8)	9.4 (2.6)	9.3 (2.7)	10.3 (1.9)
False photograph	8.3 (1.1)	8.0 (1.9)	8.8 (2.1)	9.1 (1.7)
Simple reading	10.6 (1.5)	9.9 (2.7)	10.0 (3.1)	10.9 (1.4)
**Relative motion**				
Facial Affect Matching task				
Run 1	.13 (.02)	.13 (.01)	.15 (.04)	.12 (.02)
Run 2	.13 (.03)	.13 (.02)	.16 (.04)	.13 (.02)
Run 3	.13 (.03)	.14 (.02)	.14 (.03)	.13 (.02)
Mental State Attribution task				
Run 1	.11 (.01)	.14 (.02)	.14 (.03)	.10 (.01)
Run 2	.12 (.02)	.15 (.03)	.16 (.03)	.13 (.03)
Run 3	.11 (.02)	.12 (.02)	.15 (.06)	.11 (.02)
Run 4	.13 (.02)	.14 (.03)	.15 (.04)	.13 (.02)
Run 5	.13 (.02)	.13 (.02)	.16 (.05)	.11 (.02)
Run 6	.14 (.03)	.14 (.03)	.15 (.03)	.11 (.01)
rsfMRI	.14 (.02)	.19 (.04)	.14 (.04)	.11 (.02)

Note: Values are presented as mean (SD).

### Treatment-related changes in extrinsic functional connectivity

**Fig 1:**
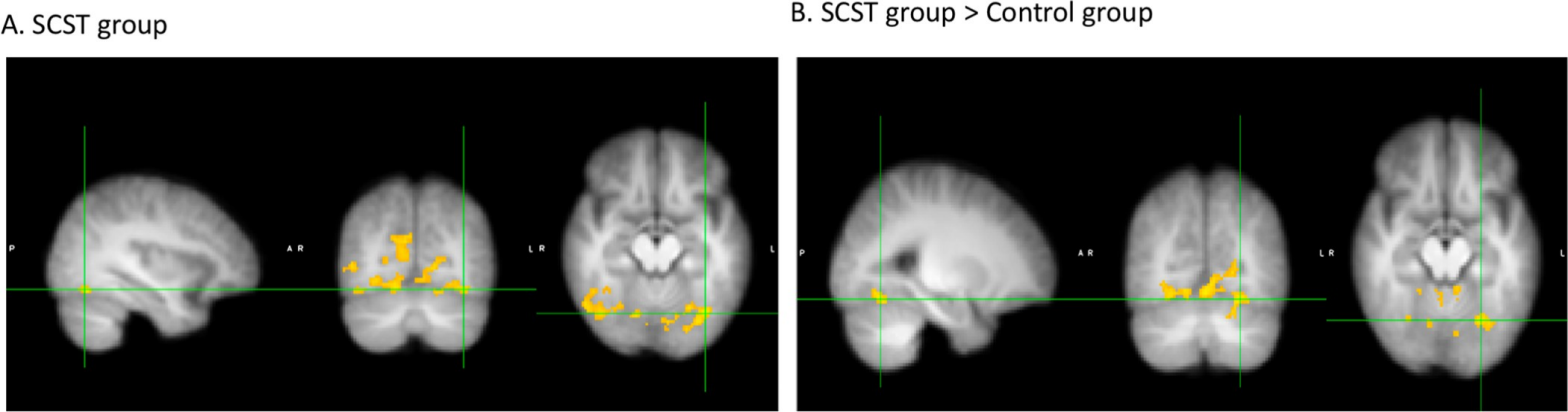
Brain areas that showed significantly increased PPI connectivity during the contrast of [emotion matching > gender matching] during the Facial Affect Matching task at 12 wks compared to baseline. A) brain areas with significant PPI connectivity in SCST group. Cross-hairs centered on MMI coordinates: x = -36, x = -68, z = -14. B) brain areas, in which SCST group showed increased PPI connectivity compared to Control group. Cross-hairs centered on MMI coordinates: x = -26, x = -64, z = -14.

For the Facial Affect Matching task, within-group comparisons indicted that the SCST group showed significantly increased PPI connectivity between the amygdala seed and several areas in the visual cortex, including the fusiform face area in relation to the contrast of [emotion matching > gender matching] (see Fig 1 and Table 3) at 12 wk compared to baseline. In the Control group, no brain region showed significant changes in PPI connectivity after the training intervention. Direct between-group comparison showed that treatment-related changes in PPI connectivity of the SCST group was greater in several areas in the visual cortex, including the fusiform gyrus (see Fig 1 and Table 3) compared to the Control group. No brain region showed greater PPI connectivity in the Control compared to the SCST group. We also did not observe any between-group difference at baseline only. Given treatment-related changes in PPI connectivity during the Facial Affect Matching Task, we explored whether PPI connectivity was associated with social functioning in the SCST group. We found that PPI connectivity between amygdala and fusiform gyrus at baseline was positively correlated with social functioning assessed with RFS at 12 wks (r = .77, p < .01), but not with social functioning at baseline. PPI connectivity at 12 wks was not associated with social functioning at 12 wks.

**Table 3:** Clusters that showed significantly increased PPI connectivity for the contrast of [emotion matching > gender matching] during the Facial Affect Matching task at 12 wks compared to baseline.

	Z statistics	x	y	z	voxels
**SCST**					
Cluster 1	3.18	-8	-74	-16	1561
Local maxima					
Lingual gyrus	3.18	-8	-74	-16	
Occipital fusiform gyrus	3.11	-36	-68	-14	
Lingual gyrus	3.04	-12	-74	-18	
Lingual gyrus	2.97	-6	-62	-4	
Lingual gyrus	2.96	16	-70	-12	
Occipital fusiform gyrus	2.95	30	-66	-8	
Cluster 2	3.21	28	-74	22	958
Local Maxima					
Lateral occipital cortex	3.21	28	-74	22	
Lateral occipital cortex	3.08	22	-76	26	
Lateral occipital cortex	2.94	-10	-86	42	
Occipital pole	2.93	-24	-94	16	
Occipital pole	2.89	-8	-96	22	
Lateral occipital cortex	2.87	10	-82	46	
**SCST > Controls**					
Cluster 1	3.13	-8	-60	-4	798
Local Maxima					
Lingual gyrus	3.13	-8	-60	-4	
Cerebella right V	3.09	14	-48	-20	
Cerebella right V	2.97	0	-64	-10	
Occipital fusiform gyrus	2.91	-26	-64	-14	
Occipital fusiform gyrus	2.84	26	-44	-22	
Lingual gyrus	2.8	-30	-68	-12	
Cluster 2	3.11	6	-74	-6	280
Local Maxima					
Lingual gyrus	3.11	6	-74	-6	
Lingual gyrus	2.96	14	-70	-8	
Lingual gyrus	2.79	28	-54	4	
Occipital fusiform gyrus	2.78	32	-62	-2	
Lingual gyrus	2.76	16	-62	-10	
Occipital fusiform gyrus	2.74	26	-64	-10	

For the Mental State Attribution task, we did not find any significant PPI connectivity related to treatment above the pre-determined threshold when examining each group separately, or when both groups were directly compared to each other. Two groups did not differ on PPI connectivity at baseline only. PPI connectivity at baseline was not correlated with social functioning at 12 wks.

### Treatment-related changes in intrinsic functional connectivity

**Fig 2:**
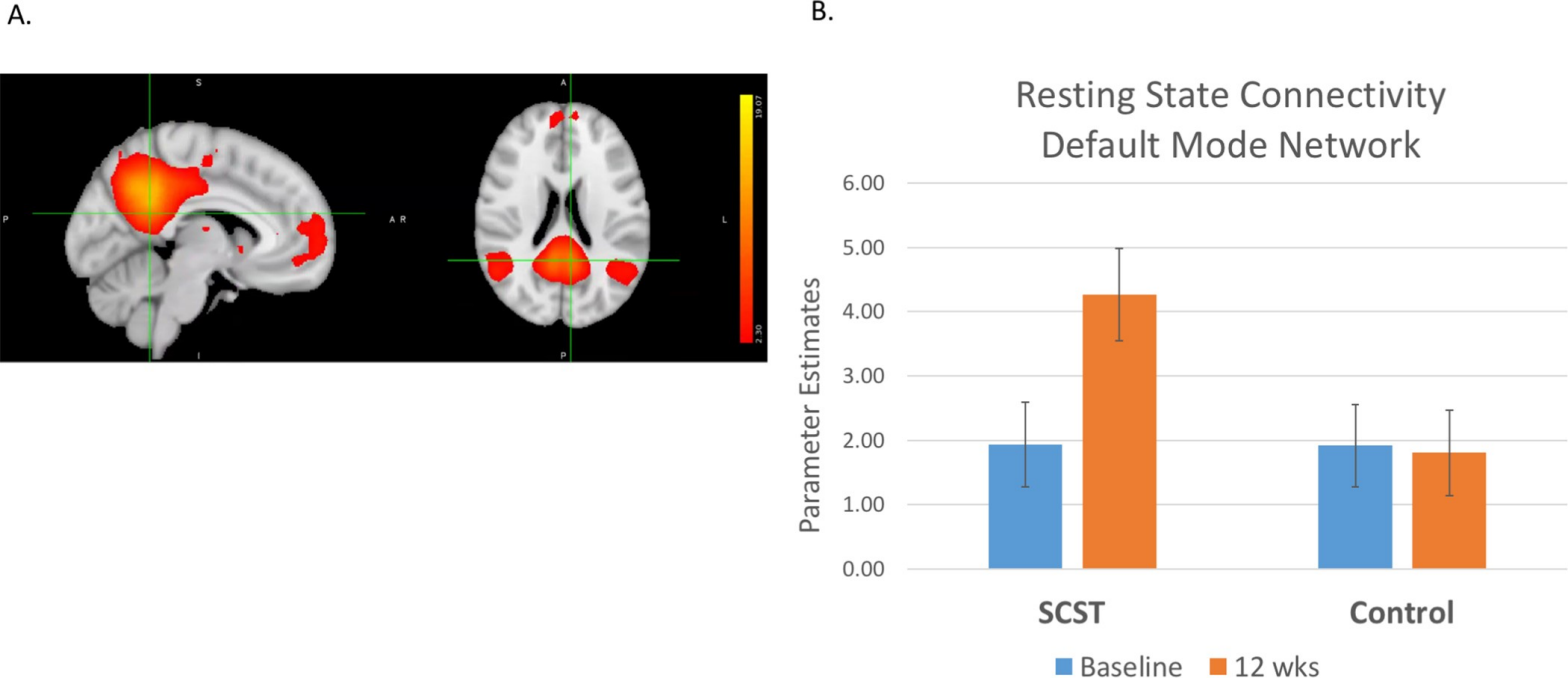
Intrinsic functional connectivity. A) default mode network component identified from rsfMRI data across groups. Cross-hairs centered on MNI coordinates: x = -6, y = -50, z = 21. B) this figure illustrates a group by interaction shown in the analysis using randomise.

For resting state network identification, the group ICA estimated 66 components. The second component, representing 2.0% percent of the explained variance, was identified as the DMN (Pearson’s *r* = .66 with the template [28]). As shown in Fig 2, the DMN consisted of medial prefrontal cortex, frontal pole, posterior cingulate gyrus, precuneus cortex, bilateral angular gyrus extending into supramarginal gyrus and superior lateral occipital cortex, and bilateral thalamus.

For the DMN there was a trend level group by time interaction (*p*_(corrected)_ = .07). The interaction was due to an increase in connectivity in the SCST group from baseline to 12 wk (*p*_(corrected)_ = .01), but no increase in the Control group (*p*_(corrected)_ = .96). Importantly, there were no between-group differences in the DMN at baseline (*p*_(corrected)_ = .21). We did not find any association between DMN connectivity and social functioning either at baseline or 12 wks in the SCST group.

## Discussion

With a focus on functional connectivity, this pilot study examined whether social cognitive skills training modulates extrinsic and intrinsic functional connectivity in psychosis. For extrinsic functional connectivity, we observed treatment-related changes in the SCST group, but not in the Control group during the Facial Affect Matching task. Specifically, the SCST group showed increased functional connectivity of amygdala with several regions in the visual cortex including fusiform face area after a 12-weeks of training. Further, the functional connectivity between amygdala and fusiform face area was positively associated with social functioning at 12-weeks. We did not observe any treatment-related changes in extrinsic functional connectivity during the Mental State Attribution task in either group. For intrinsic functional connectivity, we observed treatment-related increase in the DMN connectivity in the SCST group but not in the Control group.

While social cognitive skills intervention has been shown to improve performance of individuals with psychotic disorders on some social cognitive tasks [1, 2], little is known about brain-based biomarkers of such training effects. There have been two small published preliminary studies on fMRI-based biomarkers of social cognitive interventions in psychosis with a focus on regional activation [32, 33]. However, neither study employed a comprehensive social cognitive intervention. One study employed a computerized training that included both social and non-social components [32] and the other used a training module that focused only on facial affect recognition [33]. Further, both studies were also not able to apply rigorous correction for multiple comparisons. Thus, while both studies demonstrate feasibility, their findings were less than definitive regarding whether social cognitive skills training induces treatment-related changes at the neural level.

This study observed treatment-related changes in both extrinsic and intrinsic functional connectivity after correcting for multiple comparisons, thus providing stronger evidence that the improvements in social cognitive skills with intervention in psychosis can be observed at the neural level. Although this study had a small sample size, we recruited participants from a randomized controlled trial design and included a time- and activity-matched control training. Thus, it is unlikely that treatment-related changes in function connectivity in this study are due to nonspecific effects of skills training. The findings of this study will help guide estimation of sample size for a large randomized clinical trial to examine the robustness of the fMRI effects.

We observed treatment-related changes of extrinsic functional connectivity during the Facial Affect Matching task, but not during the Mental State Attribution task. The greater treatment-related changes in facial affect recognition is consistent with previous intervention studies on performance-based tasks. A meta-analytic review showed moderate to large treatment-related improvement (d = 0.71) on facial affect recognition and small to medium improvement (d = 0.46) on mental state attribution [2]. If treatment-related changes on mental state attribution at the neural level are also small, they might not be easily detectable with studies with a small sample size. It is also possible that 12 weeks might be too short to see any changes in functional connectivity during the Mental State Attribution.

Beyond task-specific extrinsic functional connectivity, we also found that social cognitive skills training modulated DMN during rs-fMRI. Many of the regions in the DMN are also implicated in social processing, including the medial prefrontal cortex and posterior cingulate cortex/precuneus [34]. Hence, the DMN is thought to be closely involved with various social cognitive processes, including self-related processing and mental state attribution in healthy individuals [12, 35]. Further, schizophrenia is linked to disrupted intrinsic connectivity involving DMN [36, 37], suggesting that aberrant DMN could be associated with impaired social cognition. Our finding of the effect of social cognitive skills training on DMN supports this view.

The study had several limitations. First, this study had a small sample size. While we employed appropriate statistics to control for multiple comparisons, replication with a larger sample is needed to determine the robustness of the current findings. Future studies with a larger sample could also examine whether treatment-related changes of social cognitive skills training at the neural level are related to improvement on performance-based tasks in psychosis and the sequence of changes among the symptom, functional connectivity and performance-based measures. Second, this study did not include demographically-matched healthy controls, so we do not know whether a social cognitive skills training brings the extrinsic and intrinsic functional connectivity in psychosis to normal levels. Third, chronic medicated outpatients participated in this study, and it remains unclear whether the similar levels of improvement could be observed across phase of illness. Finally, this study did not include follow-up examination of functional connectivity, so the durability of treatment-related changes remains to be determined.

To summarize, this study found that social cognitive skills training modifies extrinsic and intrinsic functional connectivity after 12-weeks of training in individuals with psychotic disorders, demonstrating that both extrinsic and intrinsic functional connectivity could be valuable biomarkers related to social cognitive skills training in psychosis. These findings suggest that treatment-related changes in functional connectivity may be critical for our understanding of underlying mechanisms of social cognitive skills training in psychosis.

## Supporting information

S1 File Supporting data is provided (ASCIfMRI_SupplementData.zip). (ZIP)
